# Improved outcomes after radiotherapy for prostate cancer: Anticoagulation, antiplatelet therapy, and platelet count as key factors in disease progression

**DOI:** 10.1002/cam4.3087

**Published:** 2020-05-13

**Authors:** Stanley I. Gutiontov, Kevin S. Choe, Jonathan L. Miller, Stanley L. Liauw

**Affiliations:** ^1^ Department of Radiation and Cellular Oncology University of Chicago Chicago IL USA; ^2^ Radiation Oncology Associates Inova Hospital Fairfax VA USA; ^3^ Department of Pathology University of Chicago Chicago IL USA

**Keywords:** blood platelets, prostatic neoplasms, radiotherapy

## Abstract

**Background:**

Several studies have suggested that antiplatelet (AP) or anticoagulant (AC) therapy may improve outcome in men with prostate cancer. We evaluated the effects of AP/AC therapy and tested the hypothesis that platelet count may also be associated with outcomes.

**Methods:**

A total of 482 patients received primary radiotherapy (median dose 72 Gy) for nonmetastatic prostate cancer; 49% received androgen deprivation therapy. NCCN risk was low/intermediate/high risk in 39%/39%/22%. AP/AC therapy and platelet counts were analyzed with respect to freedom from biochemical failure (FFBF, nadir+2), distant metastasis (FFDM), and cause specific survival (CSS).

**Results:**

After a median follow‐up of 103 months, 10‐year FFBF, FFDM, and CSS were 77%, 92%, and 96%, respectively. The 10‐year cumulative incidence of BF and DM (with death as a competing event) was 19% and 7.0%, respectively. The 32% of men on AP/AC therapy had a lower incidence of 10‐year BF (*P* = .016) and a trend toward a lower incidence of DM (*P* = .084) and CSS (*P* = .091). In the entire cohort, lowest platelet quartile (platelet count <187) was associated with higher 10‐year BF (31% vs 16%, *P* = .0042) but not DM (9.4% vs 5.2%, *P* = .22) nor CSS (*P* = .76) compared with those patients with platelet count ≥187. AP/AC therapy was associated with a larger absolute reduction in BF for men with lowest platelet quartile (10‐year BF of 21% vs 38%, *P* = .092) vs platelet ≥187 (10‐year BF of 10% vs 18%, *P* = .053). Lowest platelet quartile remained associated with higher BF and DM on multivariable analysis controlling for risk category, WBC, and Hg.

**Conclusion:**

AP/AC was associated with improved FFBF. Low platelet count was associated with inferior FFBF and FFDM after prostate radiotherapy. This association was tempered when antiplatelet and anticoagulant therapy was administered.

## Graphical abstract

482 men received primary radiotherapy for non‐metastatic prostate cancer and those men with pre‐treatment platelet count in the lowest quartile experienced worse 10‐year freedom from biochemical failure (61% vs 81%, *P* < .0001) and a trend towards worsened FFDM (88% vs 94%, *P* = .064). This negative effect was abrogated by antiplatelet therapy or anticoagulation, suggesting a connection between previously reported benefits of such therapy in prostate cancer outcomes and thrombocytopenia.

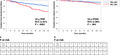

## 1 INTRODUCTION

The connection between cancer and the coagulation system is widely recognized. It has long been known that patients with cancer are prone to develop thromboembolism,^1^ and there are substantial experimental data that implicate the coagulation system in multiple cancer pathways, including tumor proliferation and metastasis.^2^, ^3^, ^4^ The use of antiplatelet (AP) and anticoagulant (AC) medications is of particular interest in prostate cancer given its high prevalence in the elderly who frequently have comorbidities requiring AP/AC therapy. Indeed, several epidemiologic and prospective studies as well as a meta‐analysis have suggested that AP/AC medications reduce the incidence of prostate cancer development,^5^, ^6^, ^7^, ^8^ while other studies have suggested a possible therapeutic effect on preexisting prostate cancer as well.

In a previous study of patients with prostate cancer treated with radiation therapy (RT), the use of AP/AC therapy was associated with higher biochemical control rates.^9^ In a subsequent study of 5955 men treated with radical prostatectomy or RT, distant metastasis and prostate cancer‐specific mortality (PCSM) were lower in men on AP/AC medications, especially in men with high risk disease. Those who were on AP/AC medication had a 10‐year PCSM of 4% as compared to 19% for men not taking such therapy.^10^

The objective of the current study was to further investigate the nature of the relationship between AP/AC therapy and prostate cancer disease outcomes. One hypothesis supported by preclinical evidence^11^ is that platelets may play a role in metastatic dissemination. We wished to test this hypothesis by exploring the interaction of various combinations of AP/AC therapy with pretreatment variables related to the hemostatic system, including but not limited to platelet count. The confirmation of previously unidentified associations between AP/AC therapy and the hemostatic system in a large dataset of prostate cancer patients would provide clinical evidence supporting or refuting evolving preclinical hypotheses. It could also potentially inform AP/AC use in the clinic for men with prostate cancer as well as the development of molecularly targeted AP/AC medications for cancer‐directed therapy.^12^, ^13^

## 2 METHODS

The current study included patients with nonmetastatic adenocarcinoma of the prostate who received primary treatment with RT at the University of Chicago from 1989 to 2006. From a cohort of 706 men, 482 men had a complete blood count with platelets drawn within 3 months before to 2 weeks after initiation of RT and comprised the study sample. Data were obtained from a database maintained with diagnostic, clinical, and pathologic information as well as patient‐reported data from questionnaires, administered at diagnosis and at regular intervals and approved for clinical research by our institutional review board.

The primary end point was freedom from biochemical failure (FFBF). Freedom from distant metastases (FFDM), cancer specific survival (CSS), and overall survival (OS) were also analyzed using the Kaplan‐Meier (1958) method.^14^ Biochemical failure was defined as prostate‐specific antigen (PSA) nadir plus 2 ng/mL^15^ and time to biochemical failure was defined as the time of the first such increase after RT completion. The sources for dates and causes of death include state‐issued death certificates and the National Death Index. Vital status and/or cause of death were available for all patients included in the current study. Life‐table product limit estimates of 10‐year rates were computed for each end point, as were cumulative incidence curves to account for competing risks.

The main independent variables of interest in this study were the use of AP/AC medications and the platelet count, which were available in all patients. Given the focus on a possible synergistic mechanism of RT with these variables, we also studied the interaction between platelet count and AP/AC use. We did this by examining the association of platelet count with outcomes stratified by AP/AC use at initiation of RT. Patients were categorized into the AP group if their medications included aspirin, clopidogrel, or other P2Y12 receptor antagonists, and they were categorized into the AC group if medications included warfarin, heparin, low‐molecular weight heparin, direct thrombin inhibitors, or other novel agents. Patients not taking any of these medications were in the reference group. Platelet count was investigated both as a continuous variable as well as by quartile. Information about blood counts was obtained from complete blood counts ± differential laboratory values.

Two main types of analyses were conducted. For analysis of the effects of AP/AC use, platelet counts, and their interaction, we estimated cumulative incidence curves and compared the 10‐year (or occasionally earlier if the last event occurred prior to 10 years) incidence of biochemical failure (BF) or distant metastases (DM). In these analyses, the death absent BF (or DM) was treated as a competing risk. We also calculated FFBF and FFDM curves via Kaplan‐Meier and used multivariable Cox (1972) proportional hazards regression models and 95% confidence intervals to estimate cause‐specific hazard ratios (HRs) for FFBF, FFDM, and CSS.^16^, ^17^ For these analyses, patients who died before the occurrence of the event of interest were censored as of the time of death. Cox regression models were also fit for the analysis of OS. Covariables potentially prognostic for the prostate cancer‐specific endpoints (FFBF, FFDM, and CSS) were selected a priori and included in all models; backward selection criteria were also used to select other covariates with a *p* value <.1 on univariate logistic regression analysis for inclusion in the multivariable models. Data were analyzed with JMP Version 14 for Windows software (SAS Institute) and Stata Version 16 (Stata Corp).

## 3 RESULTS

### 3.1 Patient characteristics

Four hundred and eighty‐two men were treated with external beam RT (EBRT), brachytherapy, or a combination of both, with or without androgen deprivation therapy (ADT). The patient characteristics and treatment details are summarized in Table 1. The median age was 69 (range, 42‐83 years). The proportions of patients with low‐, intermediate‐, and high‐risk disease were 39%, 39%, and 22%, respectively, according to the National Comprehensive Cancer Network (NCCN) Criteria.^18^ ADT was administered in 236 (49%) patients, and the majority of patients (N = 390, 81%) received EBRT monotherapy.

**TABLE 1:** Patient characteristics (n = 482)

Median age (y)	69 (range, 42‐83)
Race	
Caucasian	203 (42%)
African‐American	246 (51%)
Other/unknown	33 (7%)
Median pre‐RT PSA (ng/mL)	7.9 (range, 0.9‐242)
Clinical stage	
T1‐T2a	419 (87%)
T2b‐T2c	38 (8%)
T3‐T4	23 (5%)
Tx	0 (0%)
N0	420 (87%)
N1	5 (1%)
Nx	57 (12%)
Gleason sum	
6	271 (56%)
7	164 (34%)
8	36 (7%)
9‐10	11 (2%)
NCCN risk category	
Low	187 (39%)
Intermediate	189 (39%)
High	105 (22%)
Treatment	
EBRT	390 (81%)
Brachytherapy monotherapy	48 (10%)
EBRT + brachytherapy boost	44 (9%)
Median dose (Gy)	
EBRT	72 (range, 62‐76.4)
Brachytherapy	144 (range, 144‐145)
Brachytherapy boost	108 (range, 108‐110)
ADT	236 (49%)
Median ADT duration (mo)	4 (range, 1‐48)
Median follow‐up (mo)	103 (range, 0.9‐244)

Abbreviations: ADT, androgen deprivation therapy; EBRT, external beam radiation therapy.

Medications including AP/AC were documented at initial consultation. One hundred and fifty‐five men (32% of entire cohort) were on either AP or AC medication, with the majority of these on AP medication only (N = 120, 25%) and the remainder on AC medication alone (N = 25, 5%) or a combination of AP and AC (N = 10, 2%). The majority of men on AP medication were receiving either aspirin monotherapy or dual antiplatelet therapy, while the majority of men on AC medication were either receiving warfarin monotherapy or a combination of an AC and an AP medication (Table S1).

After a median follow‐up of 103 months, 10‐year FFBF, FFDM, and CSS were 77%, 92%, and 96% for the entire group, respectively, while the 10‐year cumulative incidence of BF and DM (with death as a competing event) was 19% and 7.0%, respectively.

### 3.2 Association of AP/AC with disease outcome

**FIGURE 1:**
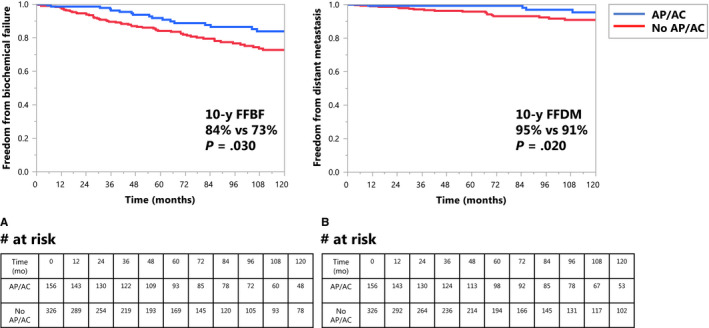
A, Kaplan‐Meier curve of freedom from biochemical failure (*P* = .030) in men taking antiplatelet therapy/anticoagulation vs not. B, Kaplan‐Meier curve of freedom from distant metastases (*P* = .020) in men taking antiplatelet therapy/anticoagulation vs not

On univariate analysis using the Kaplan‐Meier method, men taking AP/AC at time of initial consultation had improved 10‐year FFBF (*P* = .030) and 10‐year FFDM (*P* = .020) compared to those who were not on these medications. Specifically, men taking AP/AC had 10‐year FFBF of 84% vs 73% (*P* = .030) and 10‐year FFDM of 95% vs 91% (*P* = .020) (Figure 1A,B). Using cumulative incidence analysis, the 10‐year BF in men taking AP/AC was 13% vs 23% in those not taking AP/AC (*P* = .016) (Figure S1A). This was not due to a higher incidence of death prior to BF (15% vs 12%, *P* = .51). The cumulative incidence of DM was also lower among men taking AP/AC but the difference did not reach statistical significance (3.4% vs 7.6% at 9.1 years, *P* = .084) (Figure S1B). 10‐year CSS and OS in men taking AP/AC vs those who were not were 99% vs 95% (*P* = .091) and 81% vs 81% (*P* = .264), respectively.

### 3.3 Comparison of outcomes by platelet count

**FIGURE 2:**
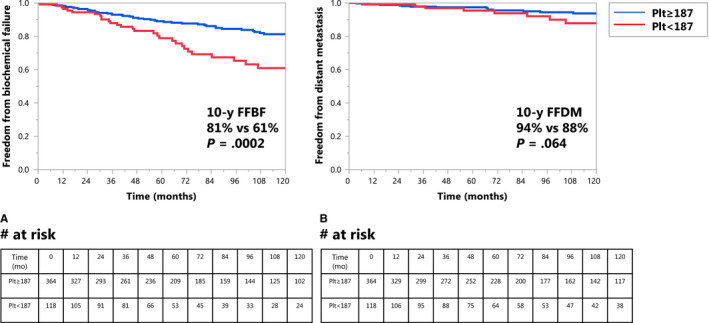
A, Kaplan‐Meier curve of biochemical failure (*P* = .0002) in men with platelet ≥187 vs <187. B, Kaplan‐Meier curve of freedom from distant metastases (*P* = .064) in men with platelet ≥187 vs <187

On logistic regression, platelet count treated as a continuous variable was associated with the occurrence of BF (*P* = .047). Platelet count stratified by median value was not associated with this outcome, but the lowest quartile (platelet count <187 000/μL, which will be abbreviated as <187) compared to the other three quartiles (quartile one 99‐186, quartile two 187‐217, quartile three 218‐256, quartile four 257‐863) was associated with higher rates of BF. There was no difference in outcome between the other platelet quartiles and there were insufficient patients with platelet count <150 to use this cutoff in the analysis. Using the Kaplan‐Meier method, outcomes in men with platelet count ≥187 vs those in the lowest quartile were as follows: 10‐year FFBF was 81% vs 61% (*P* = .0002) and 10‐year FFDM was 94% vs 88% (*P* = .064) (Figure 2A,B). Using cumulative incidence analysis, 10‐year BF was 16% vs 31% (*P* = .0042, Figure S2A). The incidence of death prior to BF was similar in the two arms. The 10‐year incidence of DM was 5.2% vs 9.4% (*P* = .22) (see Figure S2B). 10‐year CSS was 97% vs 95% (*P* = .76) and 10‐year OS was 82% vs 79% (*P* = .264). The two groups had similar NCCN risk category, radiotherapy dose and volume, baseline hemoglobin level, and ADT administration (Table 2, all *P* > .1). The only statistically significant differences between the two groups were older age (*P* = .003), shorter follow‐up (*P* = .004) and a lower white blood cell count (*P* < .0001) in the men with lowest quartile of platelets. However, leukopenia was not associated with disease outcome on univariate analysis.

**TABLE 2:** Patient characteristics, according to platelet quartile (lowest quartile vs other quartiles)

	Platelet <187 (n = 118)	Platelet ≥187 (n = 364)	*P* value
Age (median, range)	69 (42‐82)	68 (45‐83)	.003
NCCN risk category			
Low	44 (37%)	143 (39%)	.281
Medium	53 (45%)	136 (37%)	
High	21 (18%)	84 (23%)	
Initial PSA	8.2 (1.5‐106)	7.8 (1‐242)	.271
ADT use	58 (49%)	178 (49%)	.962
AP/AC use	43 (36%)	113 (31%)	.279
Hg < 12.7 (lowest quartile)	32 (27%)	78 (21%)	.223
WBC < 5.1 (lowest quartile)	48 (41%)	71 (20%)	<.001
RT dose	73.3 Gy (68.5‐76.4)	74 Gy (62‐76.4)	.496
Pelvic nodal RT	7 (6%)	27 (7%)	.545
Median follow‐up (median, range)	88 mo (0.9‐241)	107 mo (0.9‐243)	.004

Abbreviations: AC, anticoagulant; ADT, androgen deprivation therapy; AP, antiplatelet; Plt, platelet; RT, radiation therapy.

Using cumulative incidence analysis, the effect on BF of platelet quartile was most pronounced in NCCN intermediate‐and high‐risk patients. In particular, 10‐year BF in patients with platelet count ≥187 vs those in the lowest quartile was 11% vs 31% (*P* = .010) in 189 intermediate‐risk patients and 32% vs 63% at 9 years (*P* = .035) in 105 high‐risk patients.

Preplanned MVA for FFBF incorporating covariates of risk category, ADT administration, AP/AC medication use, as well as lowest quartile of platelet count, white blood cell count and hemoglobin was performed. These latter two covariates were included to minimize the potential that the observed impact of platelet count approximating thrombocytopenia was related to pancytopenia. This analysis demonstrated that both AP/AC use and platelet count <187 were associated with both FFBF and FFDM (Table 3). Of note, the cause‐specific hazard ratios for biochemical failure and distant metastasis in all men in the lowest platelet quartile relative to the other three quartiles were 2.84 and 2.60, respectively, while those associated with AP/AC use vs nonuse were 0.56 and 0.31, respectively.

**TABLE 3:** Multivariable analysis for freedom from biochemical failure and freedom from distant metastasis

	Freedom from biochemical failure		Freedom from distant metastasis	
	HR	*P* value	HR	*P* value
NCCN risk (vs low‐risk)	1.54 (Int‐risk) 5.19 (High‐risk)	<.001	2.06 (Int‐risk) 5.10 (High‐risk)	.018
Platelet <187	2.84	<.001	2.60	.025
AP/AC use	0.561	.014	0.31	.014
ADT use	1.14	.656	0.68	.454
WBC < 5.1	0.87	.563	0.67	.356
Hemoglobin < 12.7	1.13	.640	1.60	.257

Abbreviations: AC, anticoagulant; ADT, androgen deprivation therapy; AP, antiplatelet.

We also analyzed platelet to lymphocyte ratio (PLR) as a possible variable influencing outcome based on a recently published meta‐analysis demonstrating worse prostate cancer‐specific outcomes in those patients with a high PLR.^19^ We did find that PLR in the top quartile (>218) was significantly associated with poor FFBF (*P* = .047) but not with FFDM (*P* = .212). We then performed a second MVA including all cytopenias and PLR, and found that platelet count <187 remained independently associated with FFBF but not FFDM (Table S2).

### 3.4 Interaction of AP/AC medication and platelet count

**FIGURE 3:**
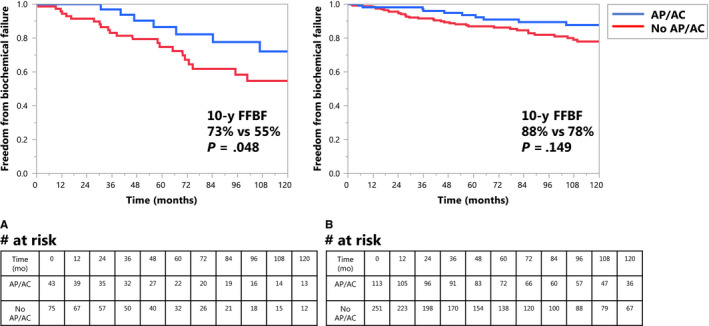
A, Kaplan‐Meier curve of freedom from biochemical failure according to antiplatelet therapy/anticoagulation use in men with platelet count <187 (*P* = .048). B, Kaplan‐Meier curve of freedom from biochemical failure according to antiplatelet therapy/anticoagulation use in men with platelet count ≥187 (*P* = .149)

To identify a potential interaction between AP/AC and low platelet count, we performed subgroup analyses using both the Kaplan‐Meier method and cumulative incidence analyses (Table S3 for cumulative incidence). Using the Kaplan‐Meier method, AP/AC use was associated with FFBF for men with platelet count <187 (Figure 3A; 10‐year FFBF 73% with AP/AC use vs 55% if no AP/AC use, *P* = .048); in men with platelet count ≥187, AP/AC use was not associated with FFBF (Figure 3B; 10‐year FFBF 88% vs 78%, *P* = .149).

Results using cumulative incidence analysis differed slightly but were generally consistent: AP/AC was associated with a trend to lower BF for men with platelet count <187 (10‐year BF 21% with AP/AC use vs 38% if no AP/AC use, *P* = .092; see Figure S3A); in men with platelet count ≥187, AP/AC use was also associated with a trend to lower BF, though the absolute numeric difference was smaller (10‐year BF 10% vs 18%, *P* = .053; see Figure S3B). On cumulative incidence analysis, AP/AC use appeared to temper the negative association of low platelet count with BF. Men not taking AP/AC had a 10‐year BF of 38% for platelet count <187% vs 18% for platelet count ≥187, *P* = .0086; men taking AP/AC had a 10‐year incidence BF of 21% for platelet count <187% vs 10% for platelet count ≥187, *P* = .17 (Table S3). Similar associations were observed for 10‐year FFDM (Kaplan‐Meier) and DM (cumulative incidence).

Patients with NCCN high‐risk disease who were not on AP/AC (n = 74) experienced a 5‐year FFBF of 71% vs 52% (*P* = .022) if their platelet count was ≥ vs <187, respectively. Men with high‐risk prostate cancer on AP/AC (n = 31) experienced a 5‐year FFBF of 74% vs 86% (*P* = .686) with the same stratification. There were no differences with regards to FFDM within these same stratifications.

We also analyzed the potential differential effects of AP medication separately from AC medication on outcomes. We first used the Kaplan‐Meier method to investigate the association between AP medication alone or AC medication alone on FFBF and FFDM and found that AP medication alone resulted in a statistically significant improvement in FFBF (*P* = .021) and a trend toward improvement in FFDM (*P* = .082). On the other hand, AC medication alone was not associated with FFBF (*P* = .428) or FFDM (*P* = .342). However, none of the 25 patients on AC therapy alone experienced DM despite long‐term follow‐up. We then performed an MVA for FFBF and FFDM with AP and AC as covariates in separate models including risk category, ADT use, and platelet <187. In the MVA model evaluating AP use, AP use was associated with FFBF (*P* = .012) and FFDM (*P* = .090), whereas there was no association between AC use and FFBF (*P* = .479) or FFDM (*P* = .212) (Tables S4 and S5).

## 4 DISCUSSION

This paper builds on a prior analysis exploring the association of AP/AC with disease outcome after treatment for prostate cancer. This study suggests that antiplatelet and anticoagulant therapy may improve outcomes particularly in those men with lower quartile platelet counts, who have a significantly worse disease outcome after RT. This association of low platelet count and outcome appears to be independent of other known clinical risk factors, is not driven by imbalances in the patient cohorts, and is not related to other cytopenias. Specifically, on MVA platelet count in the lowest quartile (<187) was associated with higher rates of biochemical and distant failure. This negative association was observed most prominently in men with high‐risk disease.

The above findings are surprising in the context of prior literature in the field and yet simultaneously serve to corroborate numerous emerging preclinical and clinical studies. Specifically, significant prior correlative data exist demonstrating that daily use of aspirin, the most commonly used antiplatelet agent, may have chemopreventive effects in prostate cancer.^20^ For example, a large cohort study of 146 113 patients showed that daily use of aspirin was associated with a reduced incidence of any malignancy and, specifically, prostate cancer among men.^21^ Furthermore, following the demonstration of improved PCSM in a large cohort of prostate cancer patients on AP medication, we had hypothesized that AP activity attenuated hematogenous metastases. This was based on a growing body of literature suggesting the pivotal role of platelet aggregation in promoting metastatic cancer cell survival and colonization at distant sites.^2^, ^11^, ^22^ In this scenario, thrombocytosis could potentially promote metastasis, and antiplatelet therapy could abrogate this effect. The significant finding in our current study of a deleterious impact of platelet count approximating *thrombocytopenia* rather than thrombocytosis on both local and distant progression in the setting of radiation therapy highlights the likely more complex interaction of platelets with the metastatic cascade. Recent advances in basic science provide potential mechanistic explanations for our findings and conversely this study provides some of the most convincing clinical evidence that these mechanisms have direct oncologic sequelae.

A recent review of platelet and megakaryocyte (MK) interactions with metastasis by Leblanc and colleagues^23^ provides several possibilities regarding the negative effects of platelet count approximating thrombocytopenia as well as the potential modulatory impact of AP/AC medications. Prior data demonstrating that platelets shelter circulating tumor cells thereby promoting survival and colonization at distant sites^22^ has been complicated by new literature demonstrating the formation of an antimetastatic niche by MKs in the bone marrow. Injection of thrombopoietin into Balb/C nude mice prior to infusion of PC3 prostate cancer cells *decreased* the extent of skeletal lesions and metastatic tumor burden despite concomitant *thrombocytosis* due to expansion of resident MKs.^24^ Directly applying this result, it is possible that a priori depletion of MKs resulting in borderline low platelet count could therefore actually increase rather than decrease the development of bone metastases and worsen FFDM in patients with prostate cancer.

An intriguing finding in our study is the impact of decreased platelet count on FFBF, suggesting that platelets may interact even earlier in tumor recurrence than at the point of clinically evident distant metastases. Animal studies have demonstrated that platelet granules are made of both pro‐proliferative (PDGF, VEGF, FGF, EGF) and antiproliferative (TSP‐1, TGF‐β, endostatin) molecules and that their make‐up can be altered by both circulating proteins as well as the MK microenvironment.^23^ Indeed, recent data have demonstrated that high expression of PDGFR‐β in prostate cancer stroma is independently associated with biochemical prostate cancer recurrence,^25^ while other lines of evidence suggest that TGF‐β released by both platelets and radiotherapy can have effects on radiosensitivity^26^ and immunity.^27^ A recent study suggests that a low platelet count may interact in novel ways in this setting by demonstrating that a low platelet environment can differentially impact platelet granule release (impacting dense granules but not alpha granules).^28^ Together, these illustrate but a few of the possible mechanistic links between platelets, prostate cancer therapy, and oncologic outcome. How exactly platelet count approximating thrombocytopenia interacts with the above framework is unclear, but we believe this warrants further study given recent publication of a population‐based analysis demonstrating increased all cancer‐specific mortality in individuals with thrombocytopenia.^29^ We further hypothesize that AP therapy may abrogate worsened FFBF and FFDM by preventing platelet activation and granule release of important downstream effectors which may include those discussed above.

While AC therapy and in particular warfarin therapy alone did not result in a statistically significant improvement in prostate cancer specific survival, the 100% rate of FFDM observed in this subset is interesting in the context of new data recently presented by Tormoen and colleagues.^30^ In their study, they demonstrated that warfarin administration to BALB/c mice at the time of radiotherapy to flank‐injected colorectal tumor models was associated with increased tumor infiltrating CD8+ lymphocytes and coexpression of CD103 as compared with those tumors treated with radiotherapy alone. They further demonstrated a trend to improved regional and distant disease‐free survival among a clinical cohort of early stage nonsmall cell lung cancer patients. Those who received radiotherapy concomitantly with warfarin fared better than those receiving radiotherapy alone. The investigators hypothesized that these effects are mediated by inhibition of MerTK, a tyrosine kinase receptor that when activated contributes to an immune tolerant tumor microenvironment via the regulation of phagocytosis by tumor‐associated macrophages. Whether or not such mechanisms play a role in prostate cancer remains to be seen and should be investigated.

Although the findings from this study may be potentially applicable to many patients diagnosed with prostate cancer, there are several caveats that need to be considered. First, because of the observational nature of this study, unforeseen interactions with other variables may have contributed to the results. Second, the dosage, duration, and timing of AP/AC use were not addressed in detail due to the retrospective nature of this study. Third, prior research has demonstrated an increased risk of clinically significant rectal bleeding in patients on AP/AC therapy who undergo prostate RT.^31^ The optimal usage of these agents, as well as the potential toxicity, should be addressed in a prospective setting. Finally, inclusion of men treated only prior to 2006, to provide sufficiently long‐term follow‐up for the maturation of prostate cancer‐specific endpoints, introduces several limitations. The first is that no patients were treated with hypofractionation or stereotactic body radiotherapy. If there is indeed a local interaction of radiotherapy, platelet count, and AP/AC medication, it is possible that fractionation may play a role in this phenomenon and therefore further study is indicated in a modern therapeutic setting. The second is that AP/AC medication has evolved in the intervening decade^32^ and this may also affect the applicability of our results to current clinical practice.

This large retrospective series demonstrates that lower platelet count is associated with increased biochemical recurrence and a twofold increased rate of DM after prostate radiation therapy. Low platelet count may serve as a surrogate for tumor‐platelet and tumor‐megakaryocyte interactions that influence disease recurrence, and the associations presented here appear to suggest that these interactions may be modifiable by existing medications of the AP/AC class.

## CONFLICT OF INTEREST

None of the authors declares a conflict of interest.

## AUTHOR CONTRIBUTIONS

SG contributed to conceptualization, data curation, formal analysis, and writing (both original draft and review and editing). KC contributed to conceptualization and review and editing. JM contributed to review and editing. SL contributed to conceptualization, formal analysis, and writing (both original draft and review and editing).

## Supporting information

Fig S1

Fig S2

Fig S3

Fig S4

Table S1‐S5

## Data Availability

The data that support the findings of this study are available on request from the corresponding author. The data are not publicly available due to privacy or ethical restrictions.
