# Supplementary figures and images for: Improved outcomes after radiotherapy for prostate cancer: Anticoagulation, antiplatelet therapy, and platelet count as key factors in disease progression
Source: Cancer Med. 2020 May 13;9(13):4667–75. doi: 10.1002/cam4.3087 (PMC7333841; doi:10.1002/cam4.3087)

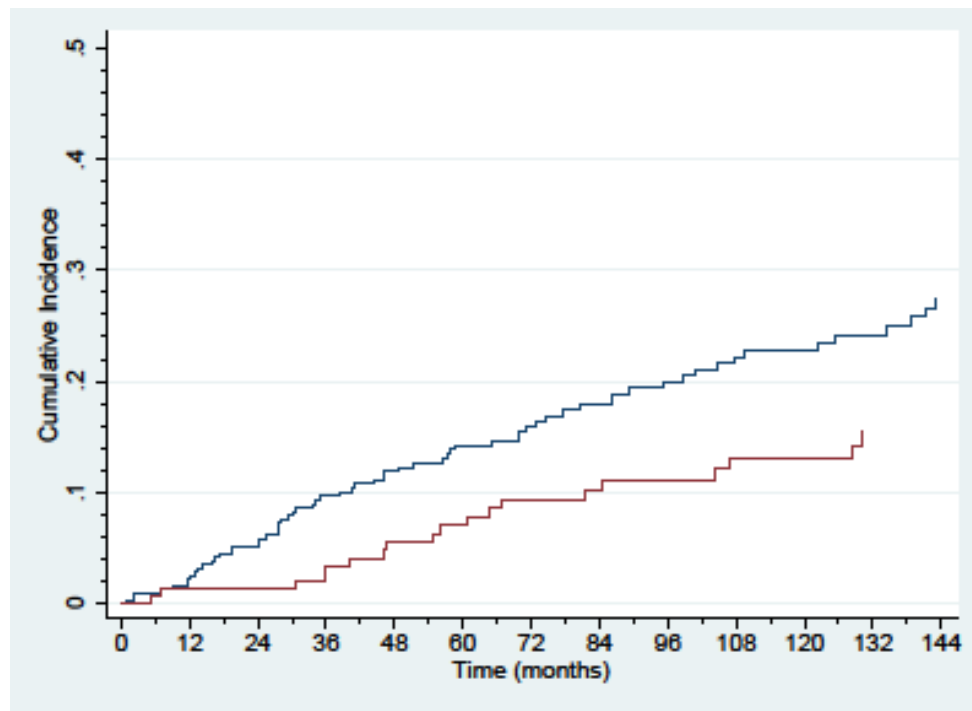

A

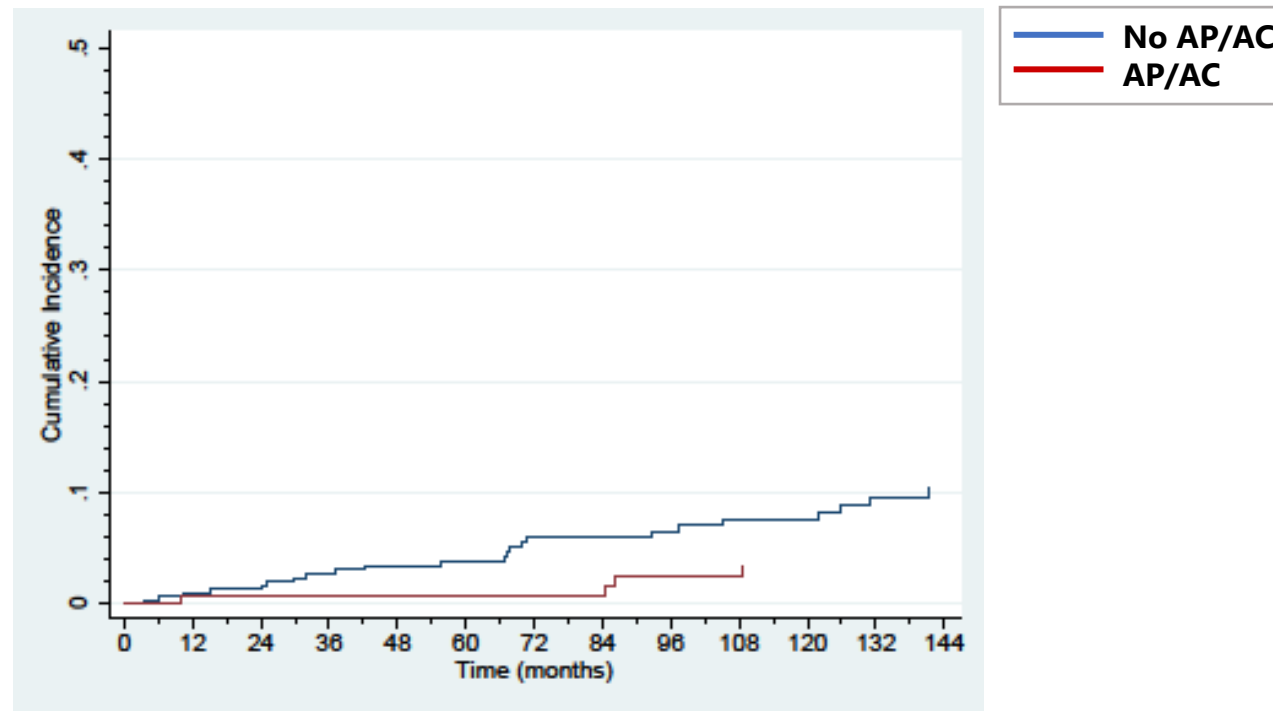

B

Supplement: Supplementary file 1 — Fig S1 [file CAM4-9-4667-s001.pdf]

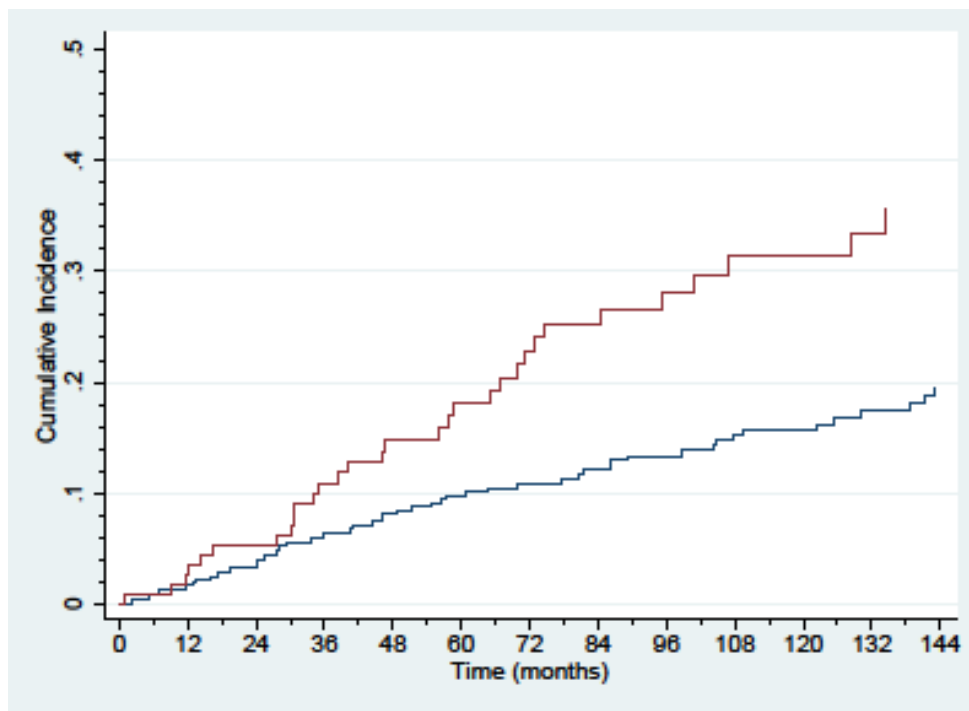

**A**

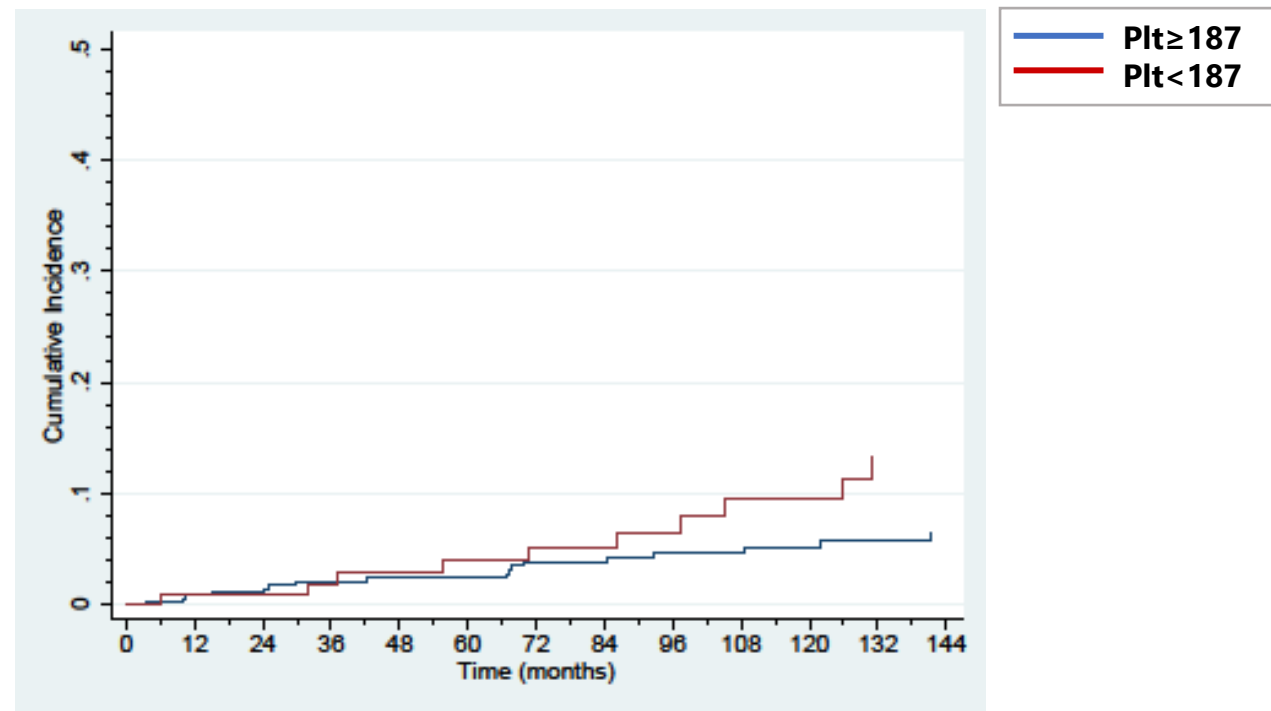

**B**

Supplement: Supplementary file 2 — Fig S2 [file CAM4-9-4667-s002.pdf]

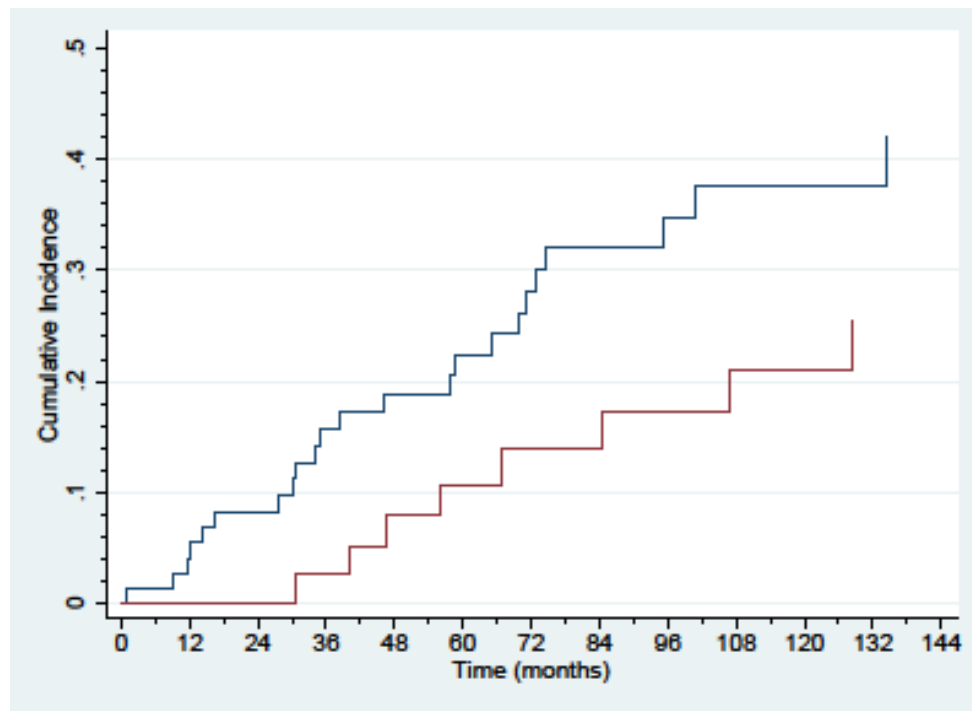

**A**

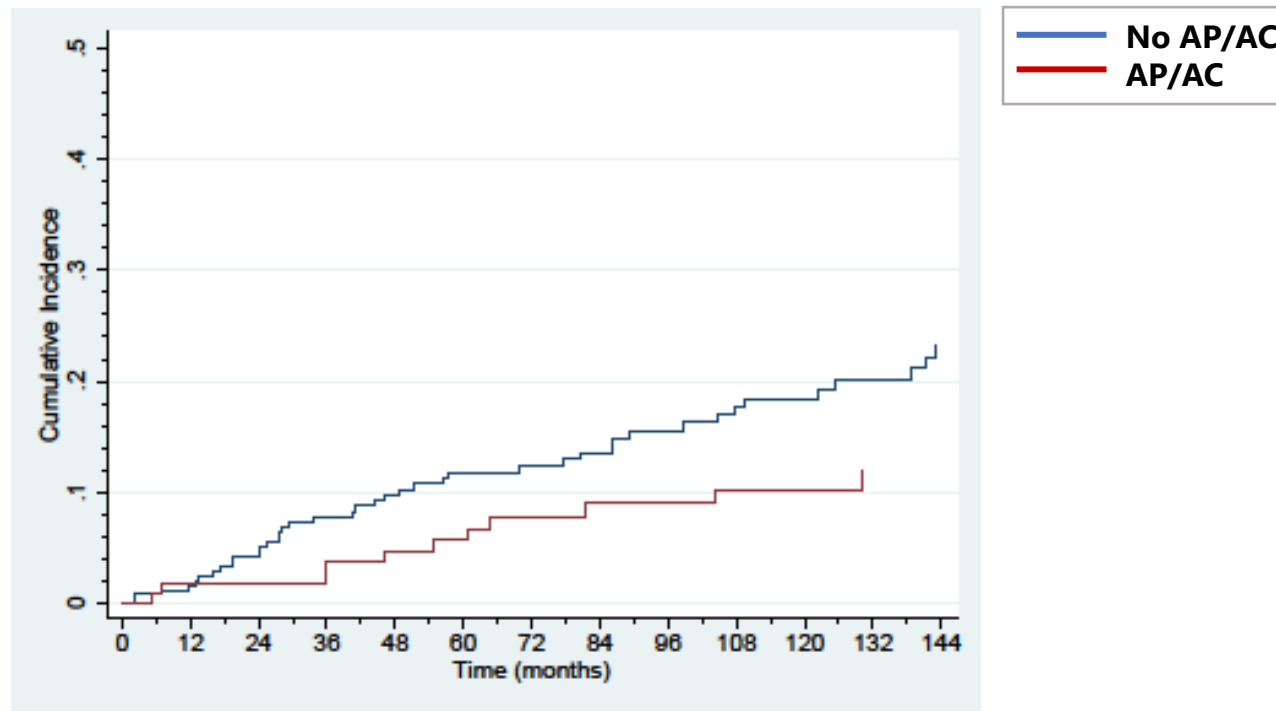

**B**

Supplement: Supplementary file 3 — Fig S3 [file CAM4-9-4667-s003.pdf]

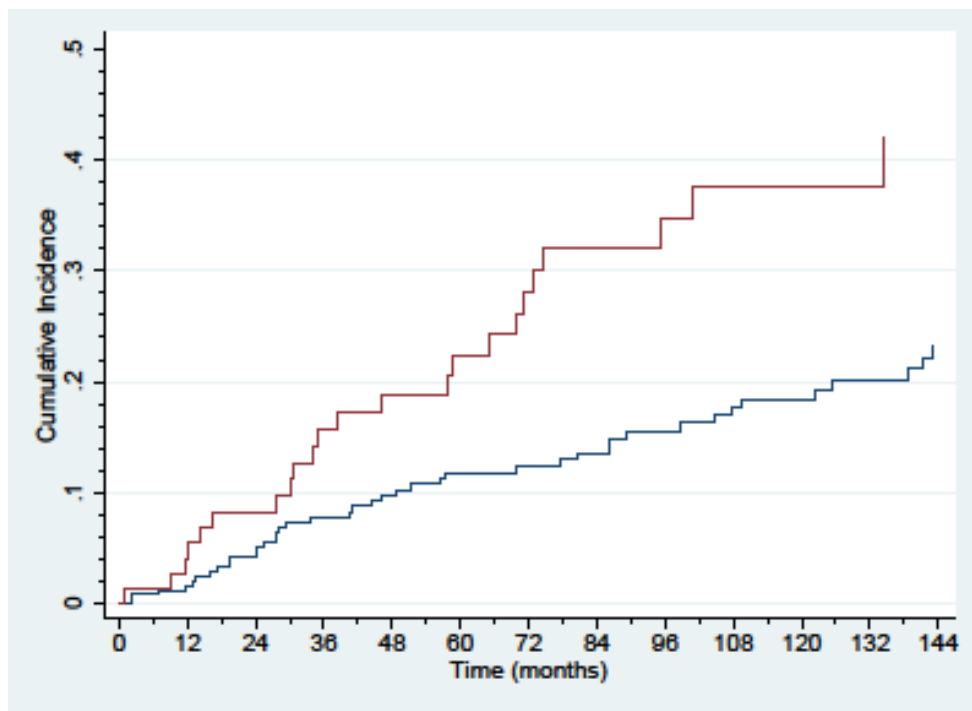

A

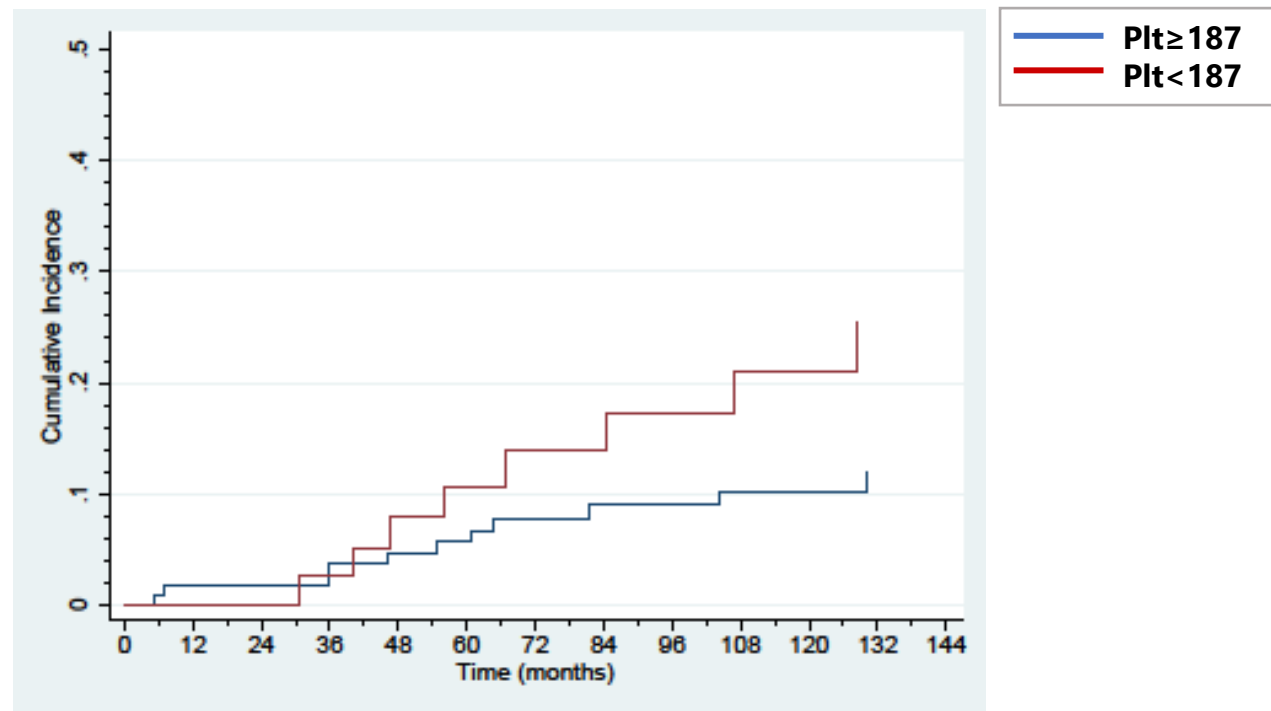

B

Supplement: Supplementary file 4 — Fig S4 [file CAM4-9-4667-s004.pdf]
