# Supplementary material for: Improved outcomes after radiotherapy for prostate cancer: Anticoagulation, antiplatelet therapy, and platelet count as key factors in disease progression
Source: Cancer Med. 2020 May 13;9(13):4667–75. doi: 10.1002/cam4.3087 (PMC7333841; doi:10.1002/cam4.3087)
Supplement: Supplementary file 5 — Table S1‐S5 [file CAM4-9-4667-s005.docx]

Supplemental Table 1. Antiplatelet and Anticoagulant Medications

|  | N (%) |
| --- | --- |
| Aspirin monotherapy | 116 (24.1%) |
| Coumadin monotherapy | 25 (5.2%) |
| Dual anti-platelet therapy | 5 (1.0%) |
| Aspirin + coumadin | 5 (1.0%) |
| Plavix monotherapy | 4 (0.8%) |

Supplemental Table 2: Multivariable analysis testing association between cytopenias, PLR, and freedom from biochemical failure and freedom from distant metastasis

|  | Freedom from biochemical failure | | Freedom from distant metastasis | |
| --- | --- | --- | --- | --- |
|  | HR | P value | HR | P value |
| Platelet < 187 | 2.27 | 0.002 | 1.75 | 0.221 |
| PLR > 218 | 1.94 | 0.018 | 1.92 | 0.157 |
| WBC < 5.1 | 0.77 | 0.345 | 0.68 | 0.411 |
| Hemoglobin < 12.7 | 1.43 | 0.189 | 2.48 | 0.034 |

PLR = platelet lymphocyte ratio (unitless); Platelet unit: 100,000 cells/uL; WBC unit: 1,000 cells/mm³; Hb unit: grams/dL

Supplemental Table 3. Cumulative incidence of biochemical failure (BF) and distant metastasis (DM).

|  | BF-10 years | P value | DM-10 years | P value |
| --- | --- | --- | --- | --- |
| AP/AC use vs non-use,  all men (n=482) | 13% vs 23% | 0.016 | 3.4% vs 7.6% | 0.084 |
| AP/AC use- vs non,  for plt<187 (n=118) | 21% vs 37% | 0.092 | 3.5% vs 8.1%^a^ | 0.35 |
| AP/AC use vs non-use,  tor plt ≥187 (n=364) | 10% vs 18% | 0.053 | 3.4% vs 6.0%^b^ | 0.33 |
| Plt <187 vs >=187,  all men (n=482) | 31% vs 16% | 0.0042 | 9.4% vs 5.2% | 0.22 |
| Plt <187 vs >=187,  AP/AC use (n=156) | 21% vs 10% | 0.17 | 3.5% vs 2.1%^a^ | 0.70 |
| Plt <187 vs >=187,  no AP/AC use (n=326) | 37% vs 18% | 0.0086 | 13% vs 6.0% | 0.16 |

AP = antiplatelet, AC = anticoagulant, Plt = platelet

^a^At 7.2 years; ^b^At 9.1 years

Supplemental Table 4: Multivariable analysis testing association between antiplatelet use only and freedom from biochemical failure and freedom from distant metastasis

|  | Freedom from biochemical failure | | Freedom from distant metastasis | |
| --- | --- | --- | --- | --- |
|  | HR | P value | HR | P value |
| NCCN Risk (vs low risk) | 1.63 (int-risk)  5.68 (high-risk) | <0.001 | 2.11 (int-risk)  5.30 (high-risk) | 0.014 |
| Platelet < 187 | 2.61 | <0.001 | 2.30 | 0.040 |
| AP only use | 0.53 | 0.012 | 0.46 | 0.090 |
| ADT use | 1.18 | 0.560 | 0.60 | 0.315 |

AP = antiplatelet, ADT = androgen deprivation therapy

Supplemental Table 5: Multivariable analysis testing association between anticoagulant use only and freedom from biochemical failure and freedom from distant metastasis

|  | Freedom from biochemical failure | | Freedom from distant metastasis | |
| --- | --- | --- | --- | --- |
|  | HR | P value | HR | P value |
| NCCN Risk (vs low risk) | 1.56 (int-risk)  5.32 (high-risk) | <0.001 | 2.18 (int-risk)  5.40 (high-risk) | 0.015 |
| Platelet < 187 | 2.51 | <0.001 | 2.22 | 0.048 |
| AC only use | 1.47 | 0.479 | NA | 0.212 |
| ADT use | 1.10 | 0.739 | 0.61 | 0.334 |

AC = anticoagulant, ADT = androgen deprivation therapy

**Supplemental Figure Legends**

Figure S1. A - Cumulative incidence of biochemical failure in men taking anti-platelet therapy / anticoagulation versus not (p=0.016 for the difference at 10 yrs); B Cumulative incidence of distant metastases in men taking anti-platelet therapy / anticoagulation versus not (p=0.084 at 9.1 yrs).

Figure S2. A - Cumulative incidence of biochemical failure in men with platelet ≥ 187 versus < 187 (p=0.0042 at 10 yrs); B - Cumulative incidence of distant metastases in men with platelet ≥ 187 versus < 187 (p=0.22 at 10 yrs).

Figure S3. A - Cumulative incidence of biochemical failure according to anti-platelet therapy / anticoagulation use in men with platelet count < 187 (p=0.092 at 10 yrs); B Cumulative incidence of biochemical failure according to anti-platelet therapy / anticoagulation use in men with platelet count ≥ 187 (p=0.053 at 10 yrs).

Figure S4. A - Cumulative incidence of biochemical failure according to platelet count in men not on anti-platelet therapy/anticoagulation use (p=0.0086 at 10 yrs). B - Cumulative incidence of biochemical failure according to platelet count in men on anti-platelet therapy/anticoagulation use (p=0.17 at 10 yrs).
